# Predictive and prognostic value of PET/CT imaging post-chemoradiotherapy and clinical decision-making consequences in locally advanced head & neck squamous cell carcinoma: a retrospective study

**DOI:** 10.1186/s12885-016-2147-y

**Published:** 2016-02-17

**Authors:** Ryul Kim, Chan-Young Ock, Bhumsuk Keam, Tae Min Kim, Jin Ho Kim, Jin Chul Paeng, Seong Keun Kwon, J. Hun Hah, Tack-Kyun Kwon, Dong-Wan Kim, Hong-Gyun Wu, Myung-Whun Sung, Dae Seog Heo

**Affiliations:** Department of Internal Medicine, Seoul National University Hospital, 101 Daehak-ro, Jongno-gu, 110-744 Seoul, Korea; Cancer Research Institute, Seoul National University College of Medicine, Seoul, Korea; Department of Radiation Oncology, Seoul National University Hospital, Seoul, Korea; Department of Nuclear Medicine, Seoul National University Hospital, Seoul, Korea; Department of Otorhinolaryngology, Seoul National University Hospital, Seoul, Korea

**Keywords:** Head and neck cancer, PET/CT, Salvage surgery, Concurrent chemoradiotherapy, Survival

## Abstract

**Background:**

The accuracy of ^18^F-fluorodeoxygluocose positron emission tomography/computed tomography (PET/CT) in predicting immediate failure after radical chemoradiotherapy (CRT) for HNSCC is poorly characterized at present. The purpose of this study was to examine PET/CT as a predictive and prognostic gauge of immediate failure after CRT and determine the impact of these studies on clinical decision making in terms of salvage surgery.

**Methods:**

Medical records of 78 consecutive patients receiving radical CRT for locally advanced HNSCC were reviewed, analyzing PET/CTs done before and 3 months after CRT. Immediate failure was defined as residual disease or locoregional and/or systemic relapse within 6 months after CRT.

**Results:**

Maximum standard uptake value (SUV) of post CRT PET/CT (postSUVmax) was found optimal for predicting immediate failure at a cutpoint of 4.4. Sensitivity, specificity, negative predictive value (NPV), and positive predictive value (PPV) were 90.0 %, 83.8 %, 98.3 %, and 45.0 %, respectively. Of 78 patients studied, postSUVmax ≥4.4 prevailed in 20 (25.6 %), with postSUVmax <4.4 in 58 (74.4 %). At postSUVmax ≥4.4 (vs. postSUVmax <4.4) OS was poorer by comparison (3-year OS: 56.9 vs. 87.7 %; *P* = 0.005), as was progression-free survival (3-year PFS: 42.9 vs. 81.1 %; *P* < 0.001). At postSUVmax ≥4.4, OS with and without immediate salvage surgery did not differ significantly (3-year OS: 60.0 vs. 55.6 %; Log-rank *P* = 0.913).

**Conclusion:**

Post CRT PET/CT imaging has prognostic value in terms of OS and PFS and is useful in predicting immediate therapeutic failure, given its high NPV. However, OS was not significantly altered by early salvage surgery done on the basis of post CRT PET/CT findings.

**Electronic supplementary material:**

The online version of this article (doi:10.1186/s12885-016-2147-y) contains supplementary material, which is available to authorized users.

## Background

More than a half million people are diagnosed with head and neck squamous cell carcinoma (HNSCC) each year, accounting for nearly 10 % of cancers worldwide [1]. Despite recent progress in treating this disease, a substantial number of patients experience locoregional and/or systemic failure (LRSF) within the first 3 years of definitive therapy [2, 3]. The prognosis with such failure is poor, marked by median overall survival (OS) <1 year [4]. Hence, there is clinical need for more timely detection of disease relapse or progression, enabling early intervention while disease burden is low.

Functional imaging with ^18^F-fluorodeoxyglucose (FDG) positron emission tomography/computed tomography (PET/CT) after definitive chemoradiotherapy (CRT) has been investigated as a useful means of detecting residual disease or recurrences earlier [2, 3]. Furthermore, PET/CT imaging has been contemplated as a source of prognostic biomarkers in HNSCC after definitive CRT [5, 6]. Despite the cumulative corroborative data that exists, use of PET/CT for this purpose remains contentious, primarily due to the lack of prospective trials that address the ramifications for patient management.

Salvage surgery is considered the most curative intervention for residual or recurrent disease in the aftermath of definitive CRT [7]. However, given the functional disability that generally results, selecting candidates appropriate for salvage surgery is often difficult. In addition, indications for salvage surgery and the survival benefits thereof are still anecdotal due to a limited body of evidence.

In the course of this study, we evaluated the predictive and prognostic value of PET/CT imaging in the context of immediate locoregional and/or systemic failure (iLRSF) after radical CRT, assessing any related impact on clinical outcomes of salvage surgery.

## Methods

### Study population

Patients treated at Seoul National University Hospital (SNUH) between January, 2005 and January, 2013 for locally advanced HNSCC (LA-HNSCC) were reviewed retrospectively. A total of 78 patients whose treatment responses were assessed by whole-body FDG PET/CT scans before and after definitive therapy qualified for study. Primary sites were oropharynx, hypopharynx, larynx, oral cavity, or nasal cavity. Biopsy-proven squamous carcinomas of unknown origin in cervical lymph nodes were presumed to be head and neck cancers and were included. Patients having more than one measurable lesion according to the Response Evaluation Criteria in Solid Tumors (RECIST) Criteria v1.1 were also admissible; and Eastern Cooperative Oncology Group performance status (ECOG PS) 0–2 was required [8]. Staging was stipulated by the American Joint Committee on Cancer (7^th^ edition).

### Treatment

Modality of radical CRT was decided through multidisciplinary approach by the SNUH Head and Neck Cancer Team. Bulky nodal status, higher T- or N-stage, and the possibility of organ preservation after induction chemotherapy influenced the decision-making process [9]. Patients of the IC/CRT group received induction chemotherapy (IC) upfront for two or three cycles every 3 weeks, followed by definitive CRT. Patients of the CRT group were given definitive CRT directly, without IC. IC regimens included docetaxel, cisplatin, 5-fluorouracil, or cetuximab. Radiotherapy was delivered daily on 5 days a week using 3-dimensional conformal radiotherapy (3D-CRT) or intensity-modulated radiotherapy (IMRT) with cisplatin or cetuximab. On planning computed tomography images, gross tumor volumes at primary sites and metastatic nodes and clinical target volumes for occult tumor spreads were delineated. Gross tumor volumes included any documented tumors in primary sites and metastatic lymph nodes with a least margin of 5 mm. Selection of cervical lymph nodal stations in clinical target volumes was decided with consideration of clinical stages, location of primary tumors and physician’s discretion. 3D-CRT was delivered using conventional fractionation with a 1.8-Gy daily dose: gross tumor received 70 Gy or higher, while high-risk and low-risk regional nodal stations received 60 Gy and 45 Gy, respectively. For IMRT, simultaneous integrated boost technique was used to deliver differential daily doses to various target volumes in 30 daily fractions: 67.5 Gy to gross tumor, 54 Gy and 48 Gy to high-risk and low-risk clinical target volumes, respectively. To account for set up errors, clinical target volumes were expanded by 3 mm to generate planning target volumes.

For second curative attempts after definitive CRT, the multidisciplinary team identified candidates for salvage surgery based on follow-up CT, MRI, PET/CT, and/or biopsy results of suspicious residual lesions. Technical feasibility and preemptive medical conditions were considered as well.

### FDG PET/CT studies

Whole-body FDG PET/CT scans were acquired before (baseline PET/CT) and 3.2 ± 1.1 months after definitive therapy (post CRT PET/CT) for early metabolic response evaluations. FDG PET/CT was done using dedicated scanners (Gemini PET/CT: Philips Healthcare, Best, The Netherlands; Biograph 40 or Biograph 64 PET/CT: Siemens Healthcare, Munich, Germany). Patients fasted for at least 6 h before FDG injection. FDG (5.18 MBq/kg) was administered intravenously, and images were acquired approximately 60 min after injection. A CT scan for attenuation correction and anatomic correlation was done first (120 kVp, 50-160 mAs). Whole-body emission scans were obtained from base of skull to proximal thigh for 2 min in recumbent position. PET images were reconstructed using iterative algorithms (ordered-subset expectation maximization) on 256 × 256 matrix.

Standard uptake values (SUVs) were calculated from the amount of injected FDG activity, body weight, and tissue uptake in the attenuation-corrected regional images as follows: SUV = (activity/unit volume) / (injected dose/body weight). For quantitative assessment of tumor FDG uptake, a spherical volume of interest (VOI) was manually drawn to include the highest radioactivity concentration of tumor or regional lymph node, using an image analysis software package (Syngo.via; Siemens Healthcare). Maximum SUV (SUVmax) was defined as the highest SUV value within the VOI range of tumor or regional lymph nodes.

### Response evaluation

Complete physical examinations and all imaging studies, including MRI or CT of head and neck and PET/CT images were assessed, as well as any CT studies (chest, abdomen) and brain MRI obtained as indicated by specific symptoms or clinical suspicions. In keeping with our institutional protocol, baseline PET/CT was done in all patients with HNSCC prior to initiation of definitive therapy. To assess response of primary tumor to CRT, CT of primary site and neck and/or MRI with contrast were performed in combination with panendoscopy at 4–8 weeks after the end of CRT as recommended in National Comprehensive Cancer Network (NCCN) guideline [10]. Most of patients underwent post CRT PET/CT 3 months after completion of definitive CRT. In some instances, post CRT PET/CT scans were done earlier or later than 3 months, as dictated by clinical suspicions of residual or recurrent disease. Follow-up imaging was performed after two or three cycles of IC, at 4–8 weeks after the end of CRT, and then every 3–6 months until progression or death. Responses to treatment were evaluated according to RECIST v1.1 [8]. Metabolic tumor response was assessed according to the SUV measurement criteria of European Organization for Research and Treatment of Cancer [11]. The metabolic complete response (mCR) was defined complete resolution of FDG uptake in the tumor such that activity is less intense than the liver and indistinguishable from surrounding background blood pool levels.

### Outcome measurement

iLRSF was defined as residual disease or locoregional and/or systemic relapse within 6 months after CRT, because in such case HNSCC was considered to be platinum-refractory [12]. As primary outcome measures, accuracy of post CRT PET/CT in predicting iLRSF and prognostic value in terms of OS and progression-free survival (PFS) were evaluated. LRSF beyond 6 months was presumed independent of post CRT PET/CT findings. As secondary objective, we evaluated the survival benefit of salvage surgery performed on the basis of post CRT PET/CT, measuring OS from date of diagnosis until death or last follow-up visit (if censored). PFS was calculated from the first day of initial IC or CCRT to the date of disease progression (confirmed by imaging or biopsy), death, or last follow-up visit (if censored).

### Statistical analysis

The differences in clinicaopathologic characteristics according to whether patients achieved mCR or not were tested for significane using Mann-Whitney test for continuous variables and the chi-square test or Fisher’s exact text for categorical variables. Of the various metabolic parameters, optimal predictive indices and cutpoints were obtained via Youden index [13]. Sensitivity, specificity, positive predictive value (PPV), and negative predictive value (NPV) were estimated. OS and PFS in all patients and in various subgroups, namely those defined by predictive thresholds, were estimated through Kaplan-Meier method. Between-group differences in OS and PFS were compared using log-rank test. All reported *P* values were two-sided, with statistical significance set at *P* < 0.05. Above calculations relied on standard software (STATA version 11; StataCorp LP, College Station, Texas, USA).

### Ethical consideration

The study was approved by the Seoul National University Hospital Institutional Review Board (SNUH IRB) (IRB approval number: H-1307-051-504) and was conducted in accordance with Declaration of Helsinki provisions. Patient informed consent was waived from SNUH IRB because of the retrospective design of the study.

## Results

### Patient characteristics

Demographic and clinical characteristics are summarized in Table 1. Median patient age was 62 years (range: 24–79 years). Induction chemotherapy (IC) was administered to a majority of patients (64.1 %). All subjects underwent PET/CT imaging at baseline, prior to initiating therapy, and after completion of CRT (median, 3.0 months; range: 0.9–6.0 months). Of the 78 patients studied, 10 (12.8 %) experienced iLRSF, confirmed by histologic examination of suspicious lesions (*n* = 5) or clinical and imaging follow-up assessments (*n* = 5). There were locoregional failures in 9 patients (11.5 %): local failure in 9 patients, both local and regional failure in 4 patients. In the remaining one patient (1.3 %), both locoregional failure and distant metastasis were documented at the time of relapse.

**Table 1:** Patient characteristics

Characteristics	Total (*N* = 78)	Non-mCR (*N* = 37)	mCR (*N* = 41)	*P*-value
Age, median (range)	62 (24–79)	62 (24–76)	61 (42–79)	0.288
Sex, *n* (%)				
Male	63 (80.8)	27 (73.0)	36 (87.8)	
Female	15 (19.23)	10 (27.0)	5 (12.2)	0.097
ECOG PS, *n* (%)				
0	19 (24.4)	8 (21.6)	11 (26.8)	
1	58 (74.4)	28 (75.7)	30 (73.2)	
2	1 (1.3)	1 (2.7)	0 (0.0)	0.601*
Location, *n* (%)				
Oropharynx	47 (60.3)	19 (51.4)	28 (68.3)	
Hypopharynx	19 (24.4)	11 (29.7)	8 (19.5)	
Larynx	3 (3.9)	2 (5.4)	1 (2.4)	
Oral cavity	5 (6.4)	4 (10.8)	1 (2.4)	
Nasal cavity/PNS	1 (1.3)	0 (0.0)	1 (2.4)	
Others	3 (3.9)	1 (2.7)	2 (4.9)	0.364*
Pathology, *n* (%)				
Undifferentiated SCC	2 (2.6)	2 (5.4)	0 (0.0)	
Poorly differentiated SCC	13 (16.7)	4 (10.8)	9 (22.0)	
Moderately differentiated SCC	20 (25.6)	7 (18.9)	13 (31.7)	
Well differentiated SCC	7 (9.0)	6 (16.2)	1 (2.4)	
Nonkeratinizing carcinoma	1 (1.3)	1 (2.7)	18 (43.9)	
Unknown/not specified SCC	35 (44.9)	17 (45.6)	0 (0.0)	0.058*
TNM Stage, *n* (%)				
III	23 (29.5)	8 (21.6)	15 (36.6)	
IVA	54 (69.2)	29 (78.4)	25 (61.0)	
IVB	1 (1.3)	0 (0.0)	1 (2.4)	0.140*
IC, *n* (%)	50 (64.1)	25 (67.6)	25 (61.0)	
FP	4 (5.1)	3 (8.1)	1 (2.4)	
DFP	16 (20.5)	6 (16.2)	10 (24.4)	
DP	26 (33.3)	14 (37.8)	12 (29.3)	
DP + cetuximab	4 (5.1)	2 (5.4)	2 (4.9)	0.544
CRT regimen, *n* (%)				
Cisplatin	71 (91.0)	33 (89.2)	38 (92.7)	
Cetuximab	3 (3.9)	1 (2.7)	2 (4.9)	
Cisplatin + cetuximab	3 (3.9)	2 (5.4)	1 (2.4)	
Cisplatin + 5-FU	1 (1.3)	1 (2.7)	0 (0.0)	0.689*
Total radiation dose				
> 60 Gy	80 (97.6)	38 (97.4)	42 (97.7)	
≤ 60 Gy	2 (2.4)	1 (2.6)	1 (2.3)	1.000*
Failure in six months, *n* (%)	10 (12.8)	10 (27.0)	0 (0.0)	<0.001*
Locoregional failure alone	9 (11.5)	9 (24.3)	0 (0.0)	
Systemic & locoregional failure	1 (1.3)	1 (2.7)	0 (0.0)	
Immediate salvage op, *n* (%)	6 (7.7)	6 (16.2)	0 (0.0)	0.009*

*Abbreviations*: *mCR* metabolic complete response, *IC* induction chemotherapy, *CRT* concurrent chemoradiotherapy, *ECOG PS* Eastern Cooperative Oncology Group performance status, *SCC* squamous cell carcinoma, *FP* 5-fluorouracil and cisplatin, *DFP* docetaxel, 5-fluorouracil and cisplatin, *DP* docetaxel and cisplatin, *5-FU* 5-fluorouracil

*Fisher’s exact test

Following CRT, 41 patients (52.6 %) qualified as mCR by post CRT PET/CT, whereas the remaining 37 patients (47.4 %) did not. However, clinicopathological characteristics of the two groups such as sex, ECOG PS, primary tumor location, pathology, TNM stage, treatment protocol, total radiation dose did not differ significantly. Although 10 patients (27.0 %) who did not achieve mCR experienced iLRSF, no patients achieving mCR suffered iLRSF (Fisher’s exact test, *P* < 0.001). Immediate salvage surgery was limited to six patients (7.7 %), none of whom achieved mCR.

### Predictive value of post CRT PET/CT compared with CT, MRI

**Fig. 1:**
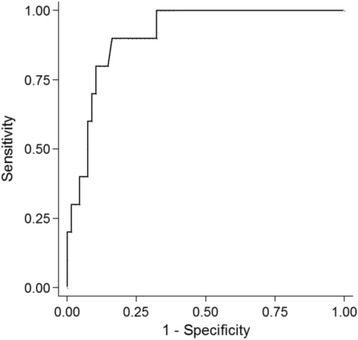
Area under reciever operating characteristic curve (AUROC) of postSUVmax predicting immediate locoregional and/or systemic failure. Abbreviations: postSUVmax, maximum standarized uptake value in PET/CT after definitive CRT

**Fig. 2:**
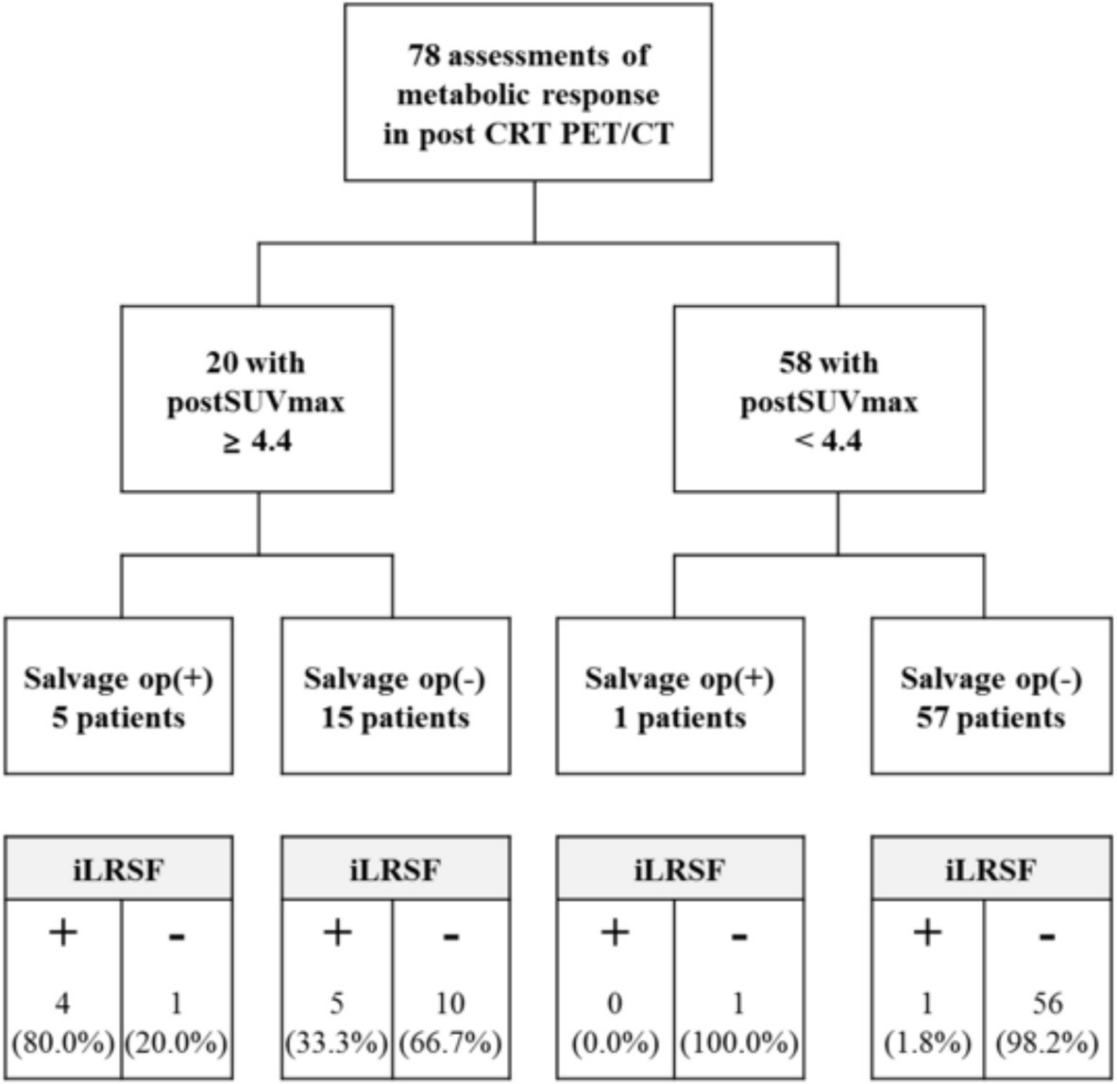
CONSORT flow of study population: Therapeutic responses in 82 patients with HNSCC were retropsectively analyzed via baseline and post CRT PET/CT images. Six of 22 patients registering postSUVmax ≥4.4 underwent salvage surgery, compared with one of 60 patients at postSUVmax <4.4. Distribution of patients experiencing iLRS is shown below by postSUVmax cutpoint (≥4.4 vs <4.4). Abbreviations: HNSCC, head & neck squamous cell carcinoma; CRT, chemoradiotherapy; PET/CT, positron emission tomography/computed tomography; postSUVmax, maximum standardized uptake value in PET/CT after definitive CRT; iLRSF, immediate locoregional and/or systemic failure

Of the various metabolic parameters, SUVmax of post CRT PET/CT (postSUVmax) best predicted iLRSF (Additional file 1: Table S1). The highest Youden index of 0.738 for postSUVmax corresponded with a cutpoint of 4.4 (Additional file 2: Table S2). The area under the receiver operating characteristic curve (AUROC) was 0.91 (95 % CI, 0.84–0.99) (Fig. 1). Of the 78 patients studied, postSUVmax ≥4.4 was documented in 20 patients (25.6 %), 9 (45.0 %) of whom experienced iLRSF; whereas in 58 patients (74.4 %) with postSUVmax <4.4, only one patient (1.7 %) experienced iLRSF (*P <* 0.001) (Fig. 2). Sensitivity, specificity, NPV, and PPV of postSUVmax were 90.0, 83.8, 98.3, and 45.0 %, respectively (Table 2).

**Table 2:** Performance of postSUVmax (at 4.4 cutpoint) and post CT or MRI in predicting immediate locoregional and/or systemic therapeutic failure

	Post PET/CT		Post CT or MRI	
	Value (%)	95 % CI	Value (%)	95 % CI
Sensitivity	90.0	55.5–99.7	44.4	13.7–78.8
Specificity	83.8	72.9–91.6	89.4	79.4–95.6
NPV	98.3	90.8–100.0	92.2	82.7–97.4
PPV	45.0	23.1–68.5	36.4	10.9–69.2

*Abbreviations*: *postSUVmax* maximum standardized uptake value in PET/CT after definitive CRT, *NPV* negative predictive value, *PPV* positive predictive value, *CI* confidence interval

For seventy five patients, CT of primary site and neck and/or MRI with contrast were performed at median 6 (range 4–12) weeks after completion of CRT. Responses to CRT were complete response (CR) in 30 (40.0 %) patients, partial response (PR) in 34 (45.3 %) patients, stable disease (SD) in 7 (9.3 %) patients and progressive disease (PD) in 4 (5.3 %) patients. Five of 64 patients who achieved CR/PR experienced iLRSF, whereas among 11 patients who did not achieve CR or PR, 4 patients experienced iLRSF (*P* = 0.023). Sensitivity, specificity, NPV and PPV of post CT or MRI were 44.4, 89.4, 92.2 and 36.4 %, respectively (Table 2).

### Survival analysis

**Fig. 3:**
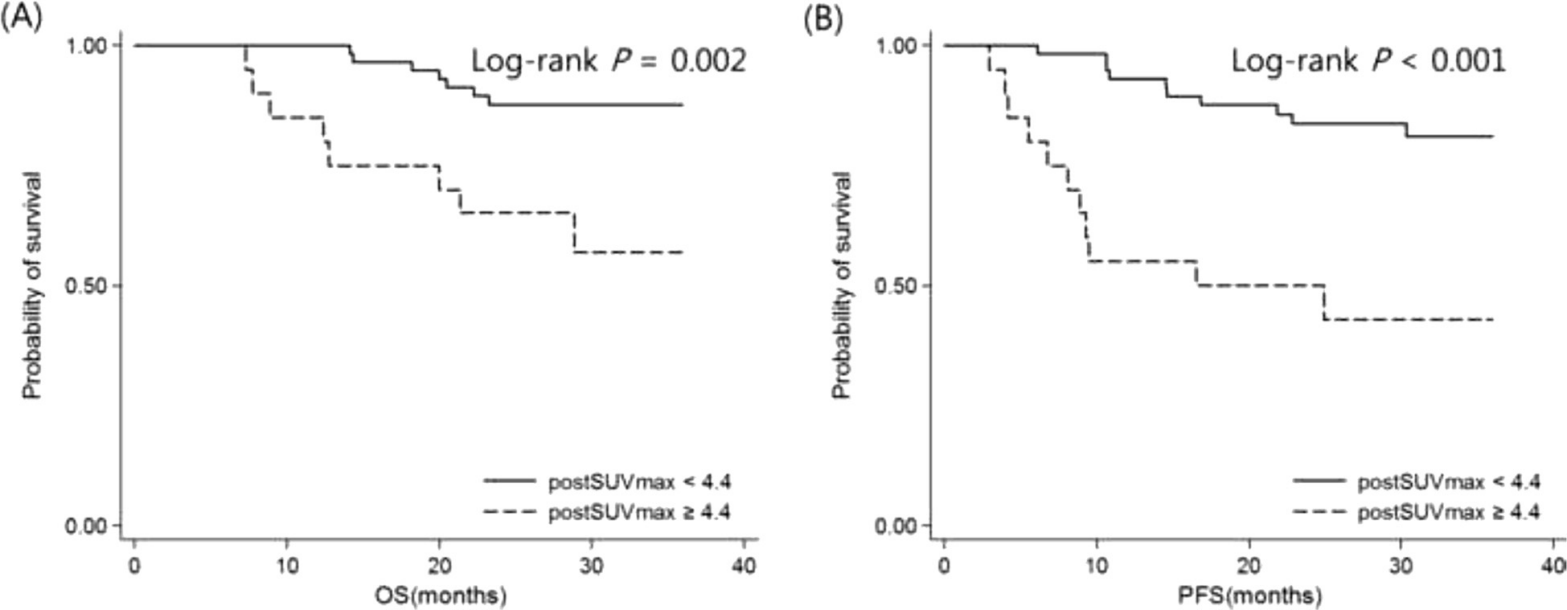
Kaplan-Meier plots of survival in LA-HNSCC patients by postSUVmax cutpoint (≥4.4 vs <4.4). OS (**a**) and PFS (**b**) were poorer in patients with postSUVmax ≥ 4.4 compared with those with postSUVmax < 4.4. Abbreviations: OS, overall survival; PFS, progression-free survival; HNSCC, head & neck squamous cell carcinoma; postSUVmax, maximum standardized uptake value in PET/CT after definitive CRT

Median follow-up duration was 52.7 months (range: 24.5–123.0 months). At postSUVmax ≥4.4 (vs postSUVmax <4.4), OS was poorer by comparison (HR = 4.25, 95 % CI: 1.54–11.74; *P* = 0.005). Three-year OS rate was 56.9 % (95 % CI, 30.1–76.3 %) in patients with postSUVmax ≥4.4, as opposed to 87.7 % (95 % CI, 75.9–94.0 %) in patients with postSUVmax <4.4 (Fig. 3a). Similarly, PFS was worse in patients with postSUVmax ≥4.4 relative to those with postSUVmax <4.4 (HR = 4.79, 95 % CI: 2.02–11.32; *P* < 0.001). Three-year PFS rates were 42.9 % (95 % CI, 20.4–63.6 %) at postSUVmax ≥4.4 and 81.1 % (95 % CI, 67.5–89.5 %) at postSUVmax <4.4 (Fig. 3b).

**Fig. 4:**
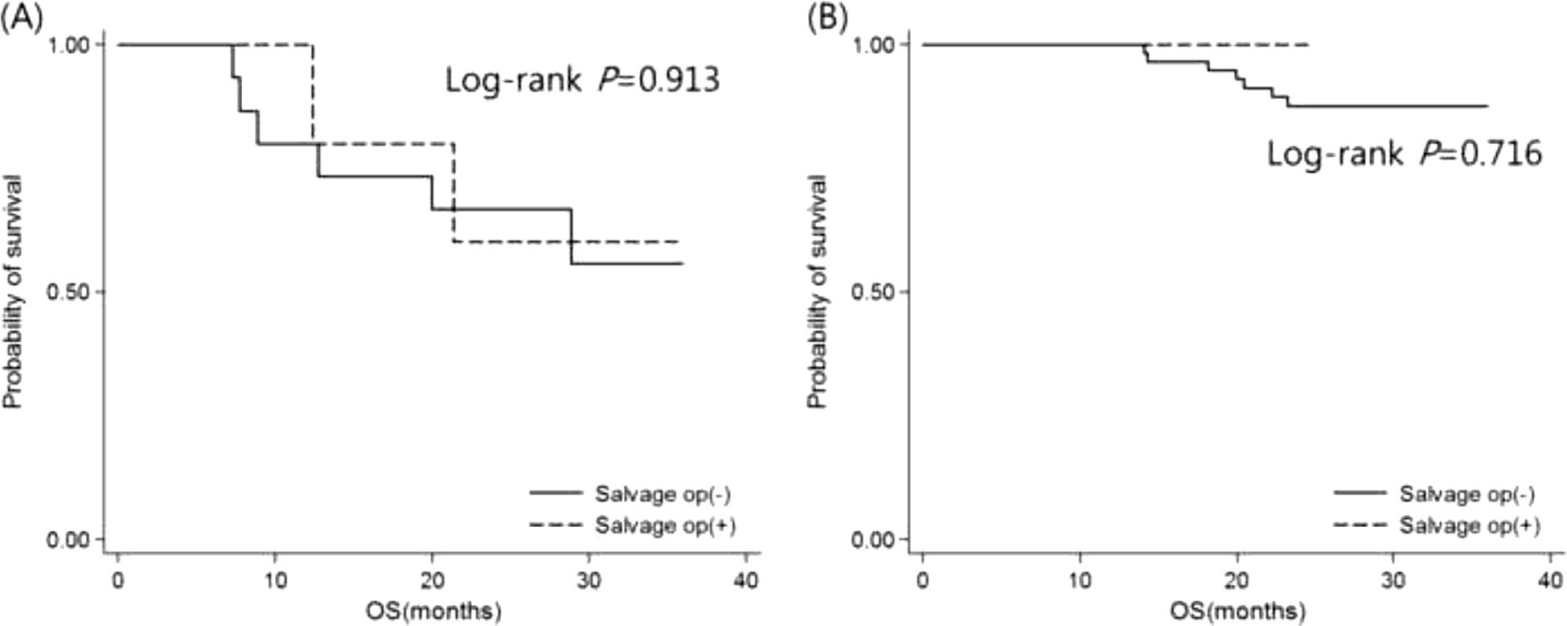
Kaplan-Meier plots of OS with and without salvage surgery in LA-HNSCC patients. OS did not differ significantly at postSUVmax ≥ 4.4 (**a**) or at postSUVmax < 4.4 (**b**). Abbreviations: OS, overall survival; HNSCC, head & neck squamous cell carcinoma; postSUVmax, maximum standardized uptake value in PET/CT after definitive CRT

Immediate salvage surgery was performed in five of 20 patients with postSUVmax ≥4.4, and one of 58 patients with postSUVmax <4.4 (Fig. 2). OS with and without immediate salvage surgery did not differ significantly at postSUVmax ≥4.4 (3-year OS: 60.0 *vs* 55.6 %; Log-rank *P* = 0.913) or at postSUVmax <4.4 (3-year OS: 100.0 *vs* 87.5 %, Log-rank *P* = 0.716) (Fig. 4).

## Discussion

Our analysis indicates that postSUVmax has value in predicting iLRSF after definitive CRT. High postSUVmax corresponded with poor prognosis, but OS was not significantly altered by early salvage surgery done on the basis of post CRT PET/CT findings.

In patients with HNSCC, recurrence is the dominant cause of treatment failure. Therefore post-treatment follow-up is well integrated into the management of HNSCC. Post-treatment PET/CT is now commonly used to gauge patient response after definitive CRT [14, 15]. Conventional imaging (including contrast-enhanced CT and MRI) has limited ability to distinguish between radiation induced inflammation or fibrosis and residual or recurrent diseases. On the other hand, PET/CT has not only improved the precision of initial staging but also yielded significantly better results for the detection of recurrence of HNSCC after CRT than CT/MRI [16]. The sensitivity, NPV and PPV of postSUVmax in our analysis were superior to those of post CT or MRI imaging when predicting iLRSF.

As discovered in previous studies, higher SUVmax values on post CRT PET/CT images may predict local recurrence and OS [17–20]. However, a decisive SUV cutpoint, enabling residual cancer to be distinguished from inflammation, has been lacking to date [21, 22]. Herein, we found that a postSUVmax cutpoint of 4.4 served well in predicting iLRSF. Furthermore, it was apparent postSUVmax also held prognostic value. In other words, OS and PFS at postSUVmax ≥4.4 (vs postSUVmax <4.4) were poorer by comparison. Within a 6-month time frame after definitive CRT, postSUVmax ≥4.4 signals the likelihood of recurrent or progressive disease. On the other hand, postSUVmax <4.4 is indicative of inflammation in the aftermath of radiation or chemotherapy.

The PPV we determined for postSUVmax was relatively low (45.0 %) yet compared favorably with the 19–58 % range reported by others [21, 23–25], and it is thought that PPV may be a function of proper timing [15]. According to Schöder et al., post CRT PET/CT should not be performed for 10–12 weeks after treatment ends [15, 22, 26]. At a lesser interval (<4-8 weeks), inflammatory changes related to radiation or chemotherapy are apt to increase false-positive interpretations. Furthermore, small-volume residual disease may escape detection by PET/CT, potentially increasing the number of false-negative readings [27]. In our cohort, 39 patients (50.0 %) viewed as high-risk for immediate failure underwent post CRT PET/CT before 3 months had elapsed, which perhaps explains the relatively low PPV of postSUVmax. Still, the true value of PET/CT imaging in this context is the high NPV attached. We recorded a remarkably high NPV (98.3 %) for postSUVmax, as did several earlier retrospective studies [21, 22, 26], suggesting that negative PET/CT scans are exceedingly reliable for determining the absence of residual disease. In fact, this strategy has helped reduced post CRT neck dissections by up to 85 % in patients initially treated for bulky nodal disease [28, 29].

If residual/recurrent disease is resectable, salvage surgery is regarded as standard of care. Nonetheless, even recent advances in extirpative and reconstructive surgical techniques have not diminished the inherent controversies. Many aspects of such surgeries are still of dubious benefit. Although studies by Bachar GY et al. and Goodwin et al. maintain that salvage surgery controls disease long-term [30, 31], other sources emphasize that these procedures (especially laryngectomy) are associated with high morbidity rates and poor overall or disease-specific survival [32, 33]. Higher rates of complications and impaired quality of life after salvage surgery have the potential to overshadow any theoretical gains [7, 32, 33]. Proper clinical selection of candidate for salvage surgery is therefore paramount. Our decision to perform salvage surgery was subsequently guided by the risk of immediate failure, based on post CRT PET/CT results. The rationale was that patients at high risk of immediate failure would benefit most. Immediate salvage surgery took place in 5 of 20 patients with postSUVmax ≥4.4, four of whom eventually developed iLRSF. Unfortunately, no significant survival benefit was demonstrated when comparing 3-year OS rates with and without aggressive surgical intervention. This disappointing outcome need to be further addressed.

Our study has a number of acknowledged limitations, the first being its retrospective design. Although a heterogenous patient population resulted, the multidisciplinary, individualized approach implemented by our institutional head and neck cancer team helped to minimize consequences. Additionally, the sample size did not confer adequate statistical power, and our index/cutpoint for predicting iLRSF may not be applicable to other institutions where patient population, equipment, and imaging protocols differ. As much as we hope our findings may prove relevant in future research, this particular investigation was never expected to be conclusive. Finally, recurrence or progression of disease in five patients was only confirmed clinically, via imaging diagnostics. This leaves some uncertainty surrounding the potential for residual viable tumor. Of note, salvage surgery, including neck dissection, is not done routinely at our institution, so opportunities for histologic verification are limited. Given that previous reports have shown a relationship between metabolic and pathologic responses [34, 35], we believe that follow-up clinical and imaging data provide reasonable support here for a disease-free state.

## Conclusions

In patients with HNSCC, functional imaging with ^18^F-fluorodeoxyglucose PET/CT after definitive CRT has prognostic value in terms of OS and PFS and is useful in predicting therapeutic response. Residual disease is effectively excluded by virtue of a high NPV. Immediate salvage surgery may also be withheld in patients achieving mCR who show no abnormal uptake on post CRT PET/CT. In our hands, however, OS was not significantly altered by early salvage surgery done on the basis of post CRT PET/CT findings. A more extensive prospective study is warranted to decide if post CRT PET/CT is acceptable as the sole or most decisive factor in managing these patients.

## Additional files

Additional file 1: Table S1. Areas under receiver operating characteristic curves of individual parameters. Additional file 2: Table S2. Youden indices of postSUVmax.

## Abbreviations

CRT: Chemoradiotherapy
HNSCC: Head and neck squamous cell carcinoma
HR: Hazard ratio
IC: Induction chemotherapy
iLRSF: Immediate locoregional and/or systemic failure
LA-HNSCC: Locally advanced head and neck squamous cell carcinoma
LRSF: Locoregional and/or systemic failure
mCR: Metabolic complete response
NPV: Negative predictive value
OS: Overall survival
PET/CT: ^18^F-fluorodeoxygluocose positron emission tomography/computed tomography
PFS: Progression-free survival
postSUVmax: Maximum standard uptake value of post chemoradiotherapy ^18^F-fluorodeoxygluocose positron emission tomography/computed tomography
PPV: Positive predictive value
SUV: Standard uptake value
