# Supplementary material for: Predictive and prognostic value of PET/CT imaging post-chemoradiotherapy and clinical decision-making consequences in locally advanced head & neck squamous cell carcinoma: a retrospective study
Source: BMC Cancer. 2016 Feb 17;16:116. doi: 10.1186/s12885-016-2147-y (PMC4756525; doi:10.1186/s12885-016-2147-y)
Supplement: Additional file 1: Table S1. — Areas under receiver operating characteristic curves of individual parameters. [file 12885_2016_2147_MOESM1_ESM.docx]

**Supplementary Table 1. Areas under receiver operating characteristic curves (AUROCs) of individual parameters**

| **Variables** | | **AUROC (95% CI)** |
| --- | --- | --- |
| SUV_base_ | Primary tumor | 0.60 (0.40-0.80) |
|  | Lymph nodes | 0.41 (0.21-0.60) |
|  | Maximum | 0.62 (0.43-0.80) |
| SUV_post_ | Primary tumor | 0.78 (0.60-0.96) |
|  | Lymph nodes | 0.81 (0.66-0.96) |
|  | Maximum | 0.91 (0.84-0.99) |
| SUV_post_ - SUV_base_ | Primary tumor | 0.37 (0.16-0.58) |
|  | Lymph nodes | 0.29 (0.15-0.42) |
|  | Maximum | 0.28 (0.10-0.47) |
| (SUV_post_ - SUV_base_) / SUV_base_ | Primary tumor | 0.23 (0.05-0.41) |
|  | Lymph nodes | 0.30 (0.06-0.53) |
|  | Maximum | 0.11 (0.03-0.19) |

Abbreviations: SUV, standardized uptake value; SUV_base_, SUV of baseline PET/CT; SUV_post_, SUV of post CRT PET/CT.
