# Supplementary material for: Predictive and prognostic value of PET/CT imaging post-chemoradiotherapy and clinical decision-making consequences in locally advanced head & neck squamous cell carcinoma: a retrospective study
Source: BMC Cancer. 2016 Feb 17;16:116. doi: 10.1186/s12885-016-2147-y (PMC4756525; doi:10.1186/s12885-016-2147-y)
Supplement: Additional file 2: Table S2. — Youden indices of postSUVmax. [file 12885_2016_2147_MOESM2_ESM.docx]

**Supplementary Table 2. Youden indices of postSUVmax**

| **postSUVmax** | **Probability of iLRSF** | **Youden index** |
| --- | --- | --- |
| 4.4 | 0.116 | 0.738 |
| 4.2 | 0.108 | 0.724 |
| 4.1 | 0.104 | 0.709 |
| 6.3 | 0.230 | 0.697 |
| 4.7 | 0.130 | 0.682 |

Abbreviations: iLRSF, immediate locoregional and/or systemic failure; postSUVmax, maximum standardized uptake value of PET/CT after definitive CRT.
